# Complement C1q expression in Erythema nodosum leprosum

**DOI:** 10.1371/journal.pntd.0006321

**Published:** 2018-03-02

**Authors:** Edessa Negera, Stephen L. Walker, Tsehaynesh Lema, Abraham Aseffa, Diana N. Lockwood, Hazel M. Dockrell

**Affiliations:** 1 London School of Hygiene and Tropical Medicine, Faculty of Infectious Tropical Diseases, London, United Kingdom; 2 Armauer Hansen Research Institute, Addis Ababa, Ethiopia; Kwame Nkrumah University of Science and Technology, GHANA

## Abstract

Complement C1q is a soluble protein capable of initiating components of the classical pathway in host defence system. In earlier qualitative studies, C1q has been implicated in the pathogenesis of Erythema Nodosum Leprosum (ENL). However, little is known about the role of this complement in ENL reaction. In the present study we described the protein level of C1q production and its gene expression in the peripheral blood and skin biopsies in patients with ENL reaction and lepromatous leprosy (LL) patient controls before and after treatment. Thirty untreated patients with ENL reaction and 30 non-reactional LL patient controls were recruited at ALERT Hospital, Ethiopia. Peripheral blood and skin biopsies were obtained from each patient before and after treatment. The level of circulating C1q in the plasma was determined by enzyme-linked immunosorbent assay. The mRNA expression of the three C1q components, C1qA, C1qB, and C1qC in the peripheral blood and skin biopsies was determined by qPCR. Circulating C1q in the peripheral blood of untreated ENL patients was significantly decreased compared to LL patient controls. Untreated ENL patients had increased C1q gene expression in the peripheral blood compared to LL controls. Similarly, C1qA and C1qC gene expression were substantially increased in the skin biopsies of untreated ENL patients compared to LL controls. However, after treatment none of these genes show significant difference in both groups. In conclusion, while circulating C1q is inversely correlated with active ENL reactions, its gene expression is directly correlated with ENL. The decreased circulating C1q may suggest the utilization of C1q in immune-complex formation in these patients. Therefore, C1q could be a potential diagnostic marker for active ENL reactions as well as for monitoring ENL treatment.

## Author summary

Erythema nodosum leprosum (ENL) is a painful inflammatory reaction which occurs in patients with lepromatous and borderline lepromatous leprosy. The diagnosis of ENL is mainly based on clinical signs and symptoms and there is no definitive laboratory diagnosis. The causes of ENL are not fully understood but immune-complexes and T-cells activation are among the main factors described by researches. Complement C1q is a protein which initiates the classical complement pathway to form antigen-antibody complex (immune-complexes) which is normally removed before deposition. However, we do not have clear information on the role of C1q in patients with ENL reaction. Therefore, we investigated the production and gene expression of C1q in patients with ENL reaction and in non-reactional LL patient controls before and after corticosteroid treatment of ENL cases. We found that the amount of circulating C-q protein was significantly reduced in untreated ENL patients compared to non-reactional LL patients and healthy controls. On the other hand, the gene expression of C1q in peripheral blood and skin biopsies had been considerably higher in untreated ENL patients compared to LL patients controls. Both C1q protein and gene expression were not significantly different between the two groups after corticosteroid treatment of ENL cases. The decreased C1q protein in untreated ENL may indicate the consumption of C1q in immune-complex formation in these patients and suggests that the involvement of immune-complex in the pathogenesis of ENL. Hence, C1q could be used as a potential candidate marker for ENL diagnose in the future.

## Introduction

Leprosy is a chronic infectious disease caused by *Mycobacterium leprae* which infects mainly skin and peripheral nerves [1]. The disease manifests with a spectrum of clinical pictures ranging from the localized tuberculoid leprosy (TT) through borderline forms to the generalized lepromatous leprosy (LL) of the Ridley-Jopling (RJ) classification [2]. Erythema nodosum leprosum (ENL) is an immune-mediated inflammatory complication affecting about 50% of patients with lepromatous leprosy (LL) and 10% of borderline lepromatous (BL) patients [3–5]. ENL first occurs as acute but may pass in to a chronic phase or can be a recurrent [6]. It involves multiple organs and manifests as systemic illness [7]. The occurrence of crops of tender erythematous skin lesion is the clinical diagnostic of ENL [8]. Histologically, the infiltrations of neutrophils throughout the dermis and subcutis is the defining characteristic of ENL[9]. However, not all clinically confirmed ENL cases have neutrophilic infiltration in the lesions.

ENL is mainly diagnosed clinically and if the facility is available supported by histological findings. Nevertheless, no clinical or laboratory tests accurately predict who is most likely to develop ENL reaction or when it might occur.

C1q is one of the several candidate markers proposed for ENL diagnosis in earlier studies [10, 11]. However, little is known about the possible role of complement in ENL reaction. Recently the complement system have been perceived as a central constituent of innate immunity, defending the host against pathogens, coordinating various events during inflammation, and bridging innate and adaptive immune responses [12]. Complement system not only protect the host against infection but also contribute to the amplification of inflammation if activated in excess or inappropriately controlled [13].

C1q is a 460-kDa protein made up of 18-subunit glycoprotein consisting of 3 subunits: A, B and C which are coded by *C1qA*, *C1qB* and *C1qC* genes respectively. It has been shown that C1q is assembled in a 1:1:1 ratio from these three different subunits. The three human C1q genes are closely located on chromosome 1 and arranged *C1qA-C1qC-C1qB* orders [14]. It is believed that C1q is mainly produced by macrophages [15]. It has been reported that IFN-γ and IL-6 promote C1q production by macrophages while IL-1 inhibits its production by macrophages [16]. C1q initiate the classical complement pathway. However, recently it has been shown that C1q is also involved in non-complement activation such as modulating dendritic cell maturation, pro-inflammatory cytokine production and T- and B-cell responses [17].

Increased C1q production in patients with active pulmonary tuberculosis (TB) compared to latent TB has recently been reported indicating the potential use of C1q as biomarker to discriminate between patients with active TB cases from latent TB [16]. C1q has also been implicated in several immune-complex disorders such as acute glomerulonephritis [18] and acute systemic lupus erythematosus (SLE) [19, 20] and rheumatoid arthritis [21].

Previous qualitative studies looked at C1q in the immunopathology of ENL and reported that serum samples from leprosy with or without reaction confer similar results[10, 11, 22]. However, serum samples from leprosy with or without reaction showed positive test. A recent microarrays study in the PBMCs of 3 ENL and 3 T1R patients has reported increased expression of the classic complement pathway particularly complement C1qA, B, C and the complement receptor C3AR1 and C5AR1 [23]. Increased fluorescent intensity of C1q in skin lesions of these patients has also been reported in the same study. Therefore, the association of C1q with the pathophysiology of ENL need to be explored in large number of ENL patient cohort. In the present study, the circulating C1q in the peripheral blood of patients with ENL and LL controls before and after prednisolone treatment was quantified using a special C1q-ELISA. The gene expression of C1q (C1qA, C1qB and C1qC) was quantified in peripheral blood and skin biopsies of these patients by quantitative polymerase chain reaction (RT-qPCR) before and after prednisolone treatment of ENL patients.

## Materials and methods

### Ethical statement

Informed written consent for blood and skin biopsies were obtained from patients following approval of the study by the Institutional Ethical Committee of London School of Hygiene and Tropical Medicine, UK, (#6391), AHRI/ALERT Ethics Review Committee, Ethiopia (P032/12) and the National Research Ethics Review Committee, Ethiopia (#310/450/06). Children under 18 years old and minor or vulnerable groups have been excluded from the study. All patient data analyzed and reported anonymously.

### Patient recruitment

We recruited 30 untreated patients with ENL reaction and 30 matched non reactional LL patient controls. All patients recruited into this study were attending the ALERT Hospital, Addis Ababa, Ethiopia. The patients were classified on the leprosy spectrum based on the Ridley-Jopling (RJ) classification schemes [2]. ENL was clinically diagnosed when a patient with BL or LL leprosy had painful crops of tender cutaneous erythematous skin lesions [4]. New ENL was defined as the occurrence of ENL for the first time in a patient with LL or BL. Lepromatous leprosy was clinically diagnosed when a patient had widely disseminated nodular lesions with ill-defined borders and BI above 2 [24]. Patients with ENL were treated according to the World Health Organization (WHO) treatment guideline with steroids that initially consisted of 40mg oral prednisolone daily and the dose was tapered by 5mg every fortnight for 24 weeks. All patients were received WHO-recommended leprosy multidrug treatment (MDT). We also included 15 apparently healthy volunteers and used for C1q ELISA result comparison only.

### Blood and skin biopsy samples

Ten micro-litter of venous blood was collected into sterile BD heparinised vacutainer tubes (BD, Franklin, Lakes, NJ, USA) before and after prednisolone treatment of ENL cases on week 24 from each patient and healthy controls. Plasma was separated and used for ELISA. In addition, 2mL of blood was collected into PAXgene Blood RNA Tubes (PreAnalytix, GmbH, Switzerland) before, and after prednisolone treatment for mRNA isolation and stored at -80°C. Six-mm punch biopsy was taken from each patient before and after prednisolone treatment into a to a Nunc tube containing 1mL RNAlater solution (Thermo-Fisher Scientific) and was kept at -20°C for 48 hours and then transferred to -80 ^o^C freezer. The ENL and LL lesions for biopsy sample were identified and marked by a dermatologist and then biopsy samples were taken from the marked area by trained research nurses under supervision. For ethical reason we didn’t collect biopsy samples from healthy controls.

### Cytokine measurement by ELISA

For quantitative detection of human C1q in the plasma samples of patients with ENL, non reactional LL and healthy controls, we used human C1q platinum ELISA (ready-to-use sandwich ELISA) with a sensitivity of 0.08ng/mL purchased from eBioscience (Affymetrix, eBioscience, UK). The procedure is briefly described as follow as: The microwell plate coated with monoclonal antibody to human C1q was aspirated and washed twice with wash buffer. Then 100μl of serially diluted C1q standard was pipetted to the first two columns of the microwell strips. To the remaining strips pre-diluted to 1:1000 plasma samples (100μl/well) were added and to the last two wells a blank (assay buffer) was added as a negative control. The plate was sealed and incubated at 22°C for 2hrs on a microplate shaker set at 400 revolutions per minute (rpm). After 2 hours incubation, the plate was washed 6 times with wash buffer and tapped on the absorbent pad. To each well, 100μl of biotin-conjugated anti-human C1q antibody was added and incubated at 22°C for 1hr on a microplate shaker set at 400rpm. Then the microplate was washed as described above and followed by the addition of 100μl Streptavidin-HRP to all wells. The plate was sealed and incubated at 22°C for 1hr on a microplate shaker as described. After six washes 100μl of TMB substrate solution was added to all wells and incubated at 22°C for 30 minutes in the dark. The colour development on the plate was monitored and the substrate reaction was stopped by pipetting 100μl of stop solution (1N H_3_PO_4_). The optical density (OD) at 450nm was measured using an ELISA plate reader (Microplate reader; Bio-Rad, Richmond, CA). A curve fit was applied to the standard curve according to the manufacturer’s manual using Microplate Manager 6 Software (Bio-Rad, Richmond, CA) and the unknown concentration of C1q in each sample was extrapolated from these standard curves.

### RNA isolation and reverse transcription

Isolation of RNA from peripheral blood and skin biopsies stored in RNAlater (Ambion, Austin, Texas) was performed using PAXgene Blood RNA Kit and RNeasy Fibrous Tissue Kit (QIAGEN Crawley, West Sussex, United Kingdom) respectively according to the manufacturer’s protocol. DNase I (QIAGEN) was included for all RNA preparations for DNA digestion. RNA yield was determined using a NanoDrop 2000, spectrophotometer (Thermo Scientific, Epsom, UK) and integrity was checked by agarose gel electrophoresis. For all samples Complementary DNA (cDNA was synthesized on the same day to avoid the risk of RNA degrades during storage. cDNA was synthesised from RNA (200 ng/reaction mixture) using High Capacity cDNA Reverse Transcriptase Kit (AB Applied Biosystems, UK). Reactions were incubated in an ABI9700 Programmable Thermal Cycler (Applied Biosystems, Foster City, California) for 10 minutes at 25°C followed by 120 minutes at 37°C and 5 minutes at 85°C and then cooling to 4°C.

### Primers and quantitative polymerase chain reaction (qPCR)

Primers between 20–24 nucleotides in length were designed across intron/exon boundaries on mRNA sequence obtained from the Nation Centre for Biotechnology Information database (NCBI) to give a product between 100-500bp. All primer sequences were blasted on the NCBI data bank to confirm their specificity. Custom synthesis of oligonucleotide primers was performed by Sigma-life science and provided in desalted form. The nucleotide sequences of the forward and reverse primers, respectively, used in this study were as follows: for C1-qA, 5'-ATGGTGACCGAGGACTTGTG-3' and 5'-GTCCTTGATGTTTCCTGGGC-3'; for C1-qB, 5'-CAGGTTGAAATCAGCATTGCC-3' and 5'-CTGTGTCAGACGCCTCCTTTC-3'; for C1-qC, 5'-AAGGATGGGTACGACGGACTG-3' and 5'- TTTCTGCTTGTATCTGCCCTC-3' and for human acidic ribosomal protein (HuPO) house-keeping gene: 5'-GGACTCGTTTGTACCCGTTG-3' and 5'-GGACTCGTTTGTA CC CG TTG-3'.

Real-time quantitative PCR for all genes was performed on the Rotor-Gene 3000 programmable thermal cycler (Corbett Life Science, Qiagen, Crawley, UK) using Roter-gene SYBR Green PCR Kit (Qiagen, Crawley, UK). The Rotor-Gene conditions were set as follows: Initial activation step (polymerase activation) was achieved by incubating at 95°C for 15 minutes, 40 cycles of denaturation at 95°C for 5 seconds, annealing at 60°C for 10 seconds, extension at 72°C for 20 seconds and fluorescence acquisition for 5 seconds at 72°C. The primer-dimer formation was checked by melting curve analysis. Melting point data were obtained by increasing the temperature from 50°C to 99°C by 1°C on each step. The interval between increases in temperature was 30 seconds for the first step and then 5 seconds for subsequent steps. An assay control was included from mRNA extraction to the amplification steps. For mRNA extraction, one assay control per batch was used. The assay control included all buffers except the sample and was processed under identical conditions with the samples. The same assay control was used during cDNA synthesis and real-time quantitative PCR.

### Calculating the relative gene expression

The relative gene expression (fold change) was analyzed by using the 2^-ΔΔ CT^ method (Cikos et al., 2007). The difference in threshold number for the amplification of the target gene (ΔC_T_) was obtained by subtracting the C_T_ of the target gene from the C_T_ of the control gene. To compare the target gene expression in patients with ENL and LL controls, ΔΔC_T_ was obtained by subtracting the ΔC_T_ of LL patient control from the ΔC_T_ of the patient with ENL. Then, the fold change (FC) was obtained by using the formula FC = 2^-ΔΔ CT^. Similarly, for the comparison of the relative target gene expression within ENL group before and after treatment, ΔΔCT was obtained by ΔC_T_ (after) minus ΔC_T_ (before). Then the fold change for the target gene expression in untreated ENL was given by 2^-ΔΔ CT^.

### Stastical methods

#### ELISA data

The optical density of each sample for each cytokine was obtained by the ELISA reader. The OD was converted to concentration (pg/mL) by microplate manager 6. Unpaired t-test was used to compare the relative concentration of the cytokines production in patients with ENL and LL controls. For comparing the cytokine concentration in patients with ENL before and after treatment, paired t-test was used. Results are presented as mean ± standard error of the mean (SE) with P-values with a cut off 0.05. SE was chosen since the primary objective of the study was to measure how the mean of the sample is related to the mean of the underlying population. SE takes standard deviation and sample size into account.

#### Real-time quantitative PCR

for the mRNA gene expression of target genes, the relative threshold cycle value (C_T_) comparison method was used. The fold change of each target gene was used for statistical analysis. Unpaired t-test was used to compare the fold change of each target gene for patients with ENL compared to LL patient controls. To compare the expression level of the desired gene in patients with ENL before and after treatment, paired t-test was used.

## Results

### Patient clinical background

Thirty patients with ENL reaction and 30 LL patient controls without ENL reaction were recruited between December 2013 and October 2015. The male to female ratio was 2:1 with a median age of 27.5 [range: 18–56] years in patients with ENL and 3:1 with a median age of 25.0 [range: 18–60] years in patients with non-reactional LL controls. All ENL patients were untreated with corticosteroid before recruitment. At time of recruitment, 20 ENL patients were previously untreated with MDT, 21 were on MDT and 5 were completed MDT treatment. Twenty non-reactional LL patients were about to start MDT, 7 were on MDT and 4 were completed MDT at recruitment. From 15 apparently healthy controls, 9 were male and 6 were female with median age of 26.5 [range: 20–57] years.

### Circulating C1q production is decreased in the peripheral blood of untreated patients with ENL reactions

**Fig 1:**
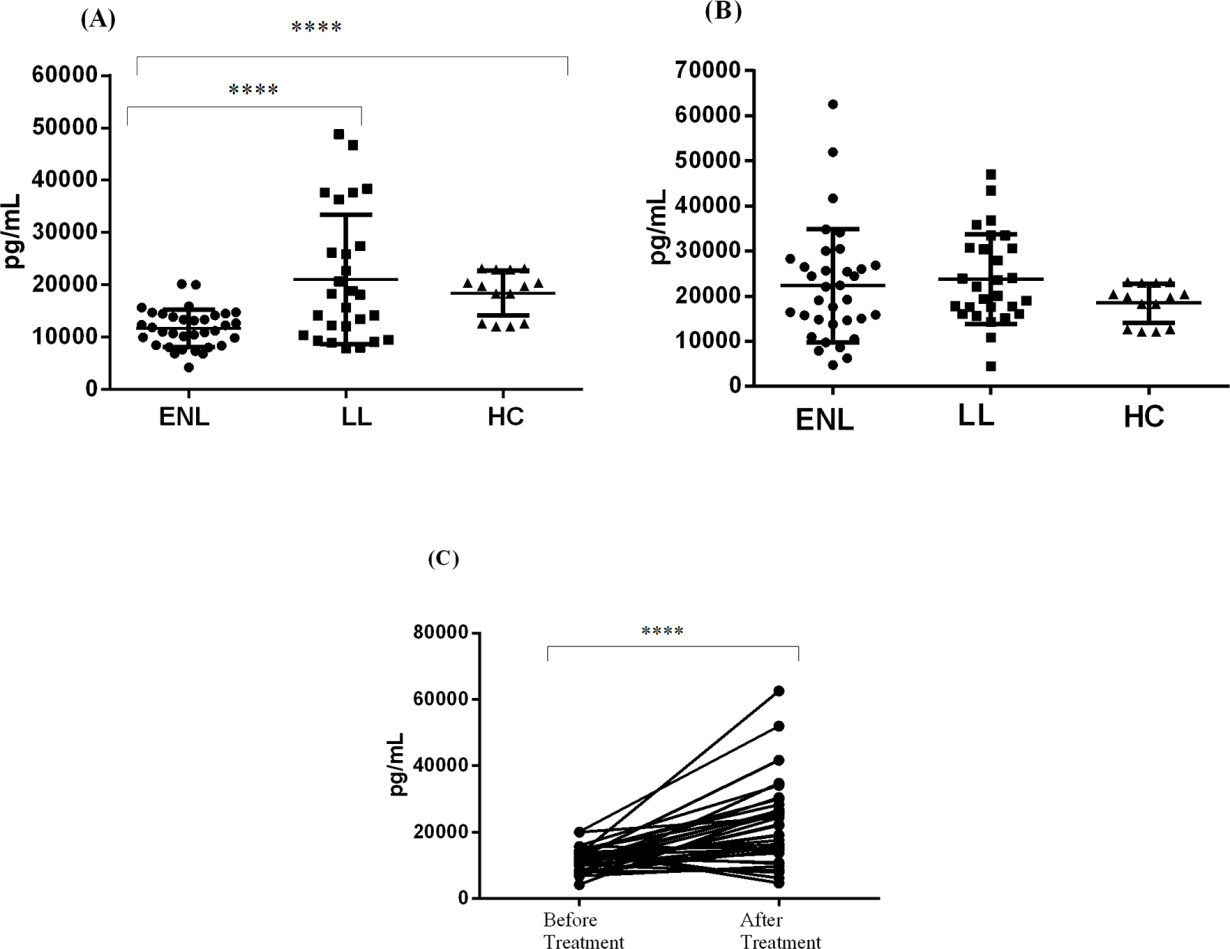
Circulating C1q production in patients with ENL, LL controls and healthy controls. (A): Before treatment, (B): after treatment, (C): within ENL groups before and after treatment. **** P < 0.*0001*. Error bars show mean ± Standard error of the mean.

Patients with ENL reactions had significantly lower circulating C1q (11698pg/mL ± 618.3) compared to LL patient controls (21059pg/mL ± 2382.0) and health controls (18448pg/mL ± 1161) before treatment (P≤ 0.0001) (Fig 1A). However, the amount of circulating C1q considerably increased in ENL patient to 22287pg/mL ± 2154 after treatment while it was only slightly increased to 23721pg/mL ± 1886 in LL patient controls. Circulating C1-q production was similar in patients with ENL and LL controls after treatment (Fig 1B). Similar, C1q production was similar in patients with ENL reactions (after treatment) and healthy controls (P≤ 0.0001). Although, C1q production was slightly higher (but not significantly different) in LL patients controls compared to health controls before and after treatment, (Fig 1B).

Comparison within patients with ENL reactions before and after treatment has shown that circulating C1q production in the peripheral blood of ENL patients was considerably increased after prednisolone treatment and the difference significantly different (P≤ 0.0001) (Fig 1C). This indicates that patients with ENL reactions do not have C1q genetic defect since the level of C1q production has increased after the ENL reactions subside.

### Increased C1q gene expression in untreated patients with ENL reactions

We quantified the mRNA gene expressions for the three genes (*C1qA*, *C1qB*, *C1qC*) in the blood and skin biopsies obtained from patients with ENL and LL controls before and after treatment.

The gene expression for *C1qA* (FC = 2.56), *C1qB* (FC = 2.63) and *C1qC* (FC = 4.55) in the peripheral blood of untreated patients with ENL reaction were significantly increased compared to the corresponding non-reactional LL patient controls (P≤ 0.05). In skin biopsies, increased expression of *C1qA* (FC = 6.45) and *C1qC* (FC = 7.46) was associated with untreated active patients with ENL reactions compared to LL patient controls (P≤ 0.05). However, unlike in the peripheral blood, the gene expression for *C1qB* in untreated patients with ENL was not statistically significantly different compared to LL patient controls. None of these genes show significant difference after treatment in both groups (Table 1).

**Table 1:** C1q gene expression in peripheral blood and skin biopsy samples from patients with ENL and LL controls before and after treatment.

Sample	gene of interest	Before treatment			After treatment		
		ΔΔC_T_	FC	P Value	ΔΔC_T_	FC	P -Value
**Blood**	C1q-A	-1.36	2.56	0.0069 *	-0.54	1.45	0.3928
	C1-B	-1.39	2.63	0.0402 *	-0.80	1.74	0.3576
	C1q-C	-2.19	4.55	0.0018 *	-0.58	1.49	0.4044
**Tissue**	C1q-A	-2.69	6.45	0.0231*	0.37	0.78	0.7661
	C1-B	0.56	0.68	0.3713	0.89	0.54	0.2072
	C1q-C	-2.90	7.46	< 0.0001 *	1.13	0.46	0.0737

Statistical test: unpaired t-test. ΔΔC_T_ = delta delta C_T_; FC = fold change

*statistically significant at α = 0.05.

C1q gene expression in the peripheral blood and skin biopsies were also compared within ENL group before and after prednisolone treatment. The gene expression for all the three genes in the peripheral blood was not statistically significantly different before and after treatment within the ENL group. The gene expression in the skin biopsies for *C1qA* and *C1qB* did not change before and after treatment within ENL group. Interestingly, the *C1qC* gene expression in the skin biopsies remarkably decreased (FC = 0.13) after treatment within ENL group (P < 0.0001) (Table 2).

**Table 2:** C1q gene expression in peripheral blood and skin biopsies samples from patients with ENL reactions before and after treatment.

Samples	Gene of interest	ΔΔC_T_	FC	P-value	Gene expression after treatment
Blood	C1q-A	0.66	0.63	0.1116	No change
	C1-B	0.64	0.64	0.3309	No change
	C1q-C	0.43	0.74	0.4178	No change
Tissue	C1q-A	-0.3	1.23	0.0839	No change
	C1-B	0.11	0.92	0.9302	No change
	C1q-C	2.93	0.13	< 0.0001 *	Decreased

Statistical test: paired t-test. ΔΔC_T_ = delta delta C_T_; Fc- Fold change

*statistically significant at α = 0.05.

## Discussion

The amount of circulating C1q in the plasma of patients with ENL and LL was quantified using a C1q ELISA. The gene expression of C1q was also quantified in blood and skin biopsy samples of these patients before and after treatment. Patients with ENL had significantly lower circulating concentrations of C1q than LL controls before treatment. However, after treatment, the amount of circulating C1q was not significantly different in both groups. This is the first work to determine circulating C1q production using ELISA in leprosy patients in leprosy patients with and without ENL. Therefore, comparison with previous studies would not have been possible. one earlier qualitative study had reported that lower C1q binding activity in the sera of untreated ENL patients compared to treated ENL patients[25].

The decreased frequency of circulating C1q in the sera of untreated patients with ENL compared to healthy controls as well as non reactional LL patient controls could be due to its utilisation by the antigen-antibody complex formation in ENL reaction. Similarly, a decreased serum complement C1q levels have been observed in other immune complex disorders such as acute glomerulonephritis (Lange et al., 1960, Lewis et al., 1971, Ohi and Tamano, 2001) and acute systemic lupus erythematosus (SLE) (Baatrup et al., 1984, Grevink et al., 2005). However, since the pathophysiology of autoimmune disorders and infectious diseases like ENL reactions are different, further study is required.

The gene expression level of *C1q A*, *B* and *C* in the blood and *C1q A*, and *C* in the skin biopsies from patients with ENL reactions were significantly higher than in LL controls before treatment. However, after treatment, none of these genes were found to be significantly different between the two groups. It is interesting to note that while the amount of circulating C1q protein is decreased in active ENL, its gene expression is increased in untreated ENL patients compared to the corresponding LL patient controls. This phenomenon may be explained by the utilization of C1q in immune-complex formation in active ENL patients. Although the initiation of ENL is not precisely known, several studies have reported the involvement of immune-complexes in the pathophysiology of ENL reaction. Immune-complexes activate the classical complement pathway that opsonise or coat antigen-antibody complexes with complement molecules which facilitates the clearance of immune-complexes by the macrophages [26]. Hence, by maintaining immune-complexes in solution, the complements allow clearance of the complexes from their site of formation, minimizing local inflammatory consequences [26]. However, in the event of immune-complexes deposition complements are consumed until [27].

In the comparison within ENL group, it has been found that C1qC gene expression was significantly increased in the skin biopsies of untreated ENL and subsequently decreased after ENL Treatment. The increased C1qC gene expiration in untreated active ENL may be associated with the immune activation in ENL reactions. Previously we have shown that increased T-cell activation in untreated active ENL patient compared to non-reactional LL controls [27]. It has also been reported that the inflammatory cytokines such as IFN-γ and IL-6 increase C1q production by macrophages [28]. Several studies indicated that C1q can modulate dendritic cell maturation, pro-inflammatory cytokine production, and T- and B-cell responses in addition to its classical function to initiate complement activation [29–31]. Recently, increased circulating C1q and *C1qC* gene expression in patients with active tuberculosis compared to healthy controls and individuals with latent TB infection has been reported [16] indicating its potential use as a biomarker to discriminate active TB from latent TB cases. Therefore, in the same scenario, *C1qC* gene expression could be used as potential diagnostic marker for ENL, which necessities further investigation particularly in the lesions.
